# Cardioprotective Effects of Qishenyiqi Mediated by Angiotensin II Type 1 Receptor Blockade and Enhancing Angiotensin-Converting Enzyme 2

**DOI:** 10.1155/2012/978127

**Published:** 2012-11-13

**Authors:** Yong Wang, Chun Li, Yulin Ouyang, Junda Yu, Shuzhen Guo, Zhongyang Liu, Dong Li, Jing Han, Wei Wang

**Affiliations:** ^1^Beijing University of Chinese Medicine, Bei San Huan Dong Lu 11, Chao Yang District, Beijing 100029, China; ^2^State Key Laboratory of Proteomics, Beijing Proteome Research Center, Institute of Radiation Medicine, Beijing 100850, China

## Abstract

The aim of this paper was to investigate whether the effects of QSYQ on CHD are associated with the renin-angiotensin-aldosterone system (RAAS). The formula groups were lavaged with QSYQ, using fosinopril sodium as a control. The level of RAAS components in the myocardial tissue was measured, respectively. The results showed that both QSYQ and fosinopril sodium can improve the ejection fraction in CHD and that QSYQ decreases the left ventricular end-systolic diameter and left ventricular end-diastolic diameter, while fosinopril sodium has no effects on these parameters. Fosinopril sodium, as an ACE inhibitor, downregulated ACE expression and eventually reduced the tissue AngII concentration but had no effect on ACE2. Moreover, it had no effect on renin or AT2, while QSYQ significantly decreased the level of renin and expression of AngII in myocardial tissue. The results also revealed that QSYQ can act on both AT1 and AT2, thus, blocking the effect of AngII and increasing the level of ACE2. It also downregulated the levels of TGF-*β* and MMP-9, but it had no effect on ACE. This study showed that the ameliorative effects of QSYQ on CHD in rats had multiple targets associated with the inhibition of RAAS, thus, producing cardioprotective therapy effects.

## 1. Introduction

Coronary heart disease (CHD) remains the single leading cause of death among adults worldwide [1]. Effective prevention and therapy for CHD pose a major challenge to the entire medical community. There exists a strong demand to continue searching for both safe and efficacious products with which to combat this emerging health epidemic. Traditional Chinese medicine (TCM) has fought against CHD and its related diseases for more than 1000 years and has accumulated thousands of herbal formulas as well as clinical studies. Some herbal formulas present a definitive clinical effect. Meanwhile, increasing numbers of patients worldwide use TCM as a complementary and alternative treatment method for CHD. 

The ancient TCM Qishenyiqi (QSYQ), prepared from a basic formula of six Chinese herbs (Radix Astragali Mongolici, salvia miltiorrhiza bunge, Flos Lonicerae, scrophularia, Radix Aconiti Lateralis Preparata, and Radix Glycyrrhizae), is widely produced in China in accordance with the China pharmacopoeia standard of quality control [2]. It is commonly used in routine treatment of CHD in clinical practice in China. It contains large-scale epidemiological survey in the randomized, controlled clinical trials proved that it has a definite effect on improving heart function [3]. In our previous study, QSYQ was shown to improve hemorheology and hemodynamics in animals with CHD [4] and suppress angiotensin II (Ang II) levels [5]. However, the mechanisms involved are poorly defined. 

Recent clinical studies have indicated that the activated renin-angiotensin-aldosterone system (RAAS) is believed to contribute significantly to the deterioration of cardiovascular function and eventually lead to myocardial remodeling. In this pathway, Ang II is considered a factor in the hypertrophy and remodeling of CHD, and it is a therapeutic target in numerous diseases, including hypertension and heart failure [6, 7]. Our previous study found that QSYQ ameliorates myocardial hypertrophy and remodeling by inhibiting the expression of Ang II in the left anterior descending coronary artery of rats. However, little is known about the exact targets of QSYQ acting on RAAS pathways in CHD. In addition to RAAS, many endothelium-derived vasoactive factors are involved in CHD treatment by regulating the vascular tone in response to a variety of stimuli. Therefore, the purpose of the present study was to investigate whether the effects of QSYQ on CHD in rats are associated with inhibition of RAAS and vital vascular endothelial regulators, such as transforming growth factor-*β* (TGF-*β*) and matrix metalloproteinase-9 (MMP-9).

## 2. Materials and Methods

### 2.1. Animals and Grouping

This study was performed in accordance with the China Physiological Society's “Guiding Principles in the Care and Use of Animals” and with approval of the Animal Care Committee of Beijing Medical Center. A total of 60 specific pathogen-free male Sprague-Dawley (SD) rats were selected (purchased from Beijing Vital River Laboratory Animal Technology Co., Ltd.) and weighed 240 ± 10 g at the start of the study.

### 2.2. CHD Model Preparation

CHD was induced by direct coronary ligation as previously described [8]. Briefly, SD rats were anaesthetized with 1% pentobarbital sodium (50 mg kg^−1^ intraperitoneally). The trachea of each rat was orally intubated with a plastic tube connected to a respirator (Kent Scientific 325, China) set at a stroke volume of 3 mL kg^−1^, respiratory ratio of 2 : 1, and rate of 80 strokes min^−1^. After left thoracotomy and exposure of the heart, the left anterior descending coronary artery was ligated with a 5-0 polypropylene suture (Surgipro, CT, USA) directly proximal to its main branching point. Sham-operated groups were prepared following an identical procedure, but without the actual tying of the polypropylene suture. The thorax was then closed, and as soon as spontaneous respiration was sufficient, the rats were extubated and allowed to recover under a heated lamp. They were fed a standard diet and water and maintained on a 12 h light and dark cycle. After ECG testing, rats that averaged QT-interval prolongation in three precordial leads were included in the study. 

The overall mortality of rats that underwent induction of myocardial infarction (MI) during the entire experimental period (up to 28 days after MI) was 30% to 40%. The majority of death occurred on the day of or the day after the MI surgery, probably because of acute pump failure or lethal arrhythmias. The rats were then randomly divided into three groups: eight in the model group, eight in the fosinopril sodium group, and eight in the QSYQ group. Meanwhile, eight rats in the sham-operated group were investigated together. The QSYQ group was treated for 28 days by daily oral gavage with a total daily dose of 2.33 g/kg of concentrated QSYQ (Beijing University of Chinese Medicine, Beijing, China) dissolved in water. The sham-operated group and model groups received the same volume of water, and the fosinopril sodium group was given the same volume of fosinopril sodium (1.2 mg/kg) via oral gavage as the QSYQ vehicle. At the end of the study, all animals were euthanized with isoflurane (Abraxis BioScience, Richmond Hill, Canada) following an overnight fast. Heart tissue samples were excised parallel to the coronary sulcus, 3 mm from the cardiac apex. All samples were immediately frozen in liquid nitrogen for further examination.

### 2.3. Echocardiographic Assessment of Left Ventricular Function

Echocardiography was used to detect the left ventricular end-systolic diameter (LVEDs), left ventricular end-diastolic diameter (LVEDd), ejection fraction (EF), fractional shortening (FS), and other indicators. A PST 65A sector scanner (8-MHz probe) was employed, which generates two-dimensional images at a frame rate of 300 to 500 frames/s. The LV dimension (LVD) was measured using m-model fractional shortening, and FS% was calculated using the following equation: FS% = [(LVEDd−LVEDs)/LVEDd] × 100. 

### 2.4. Preparation and Dose Consideration of Concentrated QSYQ

The QSYQ used in the present study was manufactured by the Beijing University of Chinese Medicine (Beijing, China) using the following six Chinese herbs: 460 g radix astragali mongolici, 230 g salvia miltiorrhiza bunge, 160 g flos lonicerae, 160 g scrophularia, 140 g radix aconiti lateralis preparata, and 90 g radix glycyrrhizae. Briefly, following extraction with 95% ethanol, the residue of Radix Astragali Mongolici was mixed with all salvia miltiorrhiza bunge, Flos Lonicerae, scrophularia, and Radix Glycyrrhizae, followed by two 2 h extractions with hot water. The water extract was then concentrated to form a paste, ethanol was added for 24 h, and the filtration was collected to form the final product. Based on the recommended daily human dosage of 20 g/d, and in accordance with the equivalent conversion between animals and people by body surface area, a dosage of 2.33 g/kg body weight was chosen in the present study.

### 2.5. Determination of Plasma Angiotensin II and Aldosterone by Radioimmunoassay

The plasma was homogenized in saline containing an enzyme inhibitor (10 *μ*L of 0.3 M EDTA-Na, 10 *μ*L of 0.34 M 8-hydroxyquinoline, and 5 *μ*L of 0.32 M dimercaptopropanol) (1 mL blood) on ice. The homogenate was centrifuged at 8000 ×g for 10 min. The supernatant was used for determination of angiotensin II (Ang II) using a radioimmunoassay kit (Beijing Kangyuan Ruide Biotechnology Co., Ltd., Beijing, China) following the manufacturer's instructions.

### 2.6. Measurement  of  Indicators  by  Western**  **Blot

The cardiac tissue was homogenized in RIPA buffer (50 mM Tris HCl, pH 7.4; 150 mM NaCl, 2 mM EDTA, 1% NP-40, and 0.1% SDS), and total protein was extracted from this homogenate. The protein concentration in each sample extract was measured using a protein assay kit (lot no. MB155207A; Pierce Co., USA) and then adjusted to the same value in all samples with 2 × 4% SDS sample buffer. The samples were boiled for 5 min followed by loading on a 12.5% SDS-PAGE gel (50 mg protein/10 *μ*L per well) for electrophoresis using a Bio-Rad mini gel apparatus at 100 V for 2 h. The fractionated protein on the gel was transferred onto an NC membrane (Beijing Pu Lilai Gene Technology Co., Ltd., Beijing, China) and electrophoresed at 300 mA for 90 min. The membrane was first probed with AT1R primary antibody (Anti-Ang II type 1 receptor (AT1) antibody, ab18801, Abcam, 1 : 500; rabbit polyclonal to Ang II type 2 receptor (AT2) antibody, ab19134, Abcam, 1 : 500; rabbit monoclonal to MMP-9, ab76003, Abcam, USA, 1 : 2000; and rabbit polyclonal to TGF-*β*1, ab92486, Abcam, USA, 1 : 500) and secondary antibody (donkey polyclonal secondary antibody to rabbit IgG-HRP, ab97064, Abcam, 1 : 5000). It was then treated with ECL (ECL Plus Western Blotting Detection Reagent; GE Healthcare) for 1 min at room temperature. The bands in the membrane were visualized and analyzed using UVP BioImaging Systems. After obtaining the AT1R (or AT2R, MMP-9, and TGF-*β*) blot density, the membrane was treated using Restore Western Blot Stripping Buffer (Thermo Scientific) to remove the AT1R (or AT2R, MMP-9, and TGF-*β*) signal, followed by probing with glyceraldehyde-3-phosphate dehydrogenase (GAPDH) primary antibodies (GAPDH mouse monoclonal IgG, ab8245, Abcam, 1 : 2000) using the same process as that used for the AT1R antibody to obtain the AT1R and GAPDH blot densities. The final reported data are the normalized AT1R band densities by GAPDH.

### 2.7. Measurement of Indicators by Immunohistochemistry

An avidin-biotin-peroxidase complex commercial method (Cell & Tissue Staining Kit; R&D Systems, Inc., USA) was used for immunohistochemistry. Briefly, 4-mm-thick paraffin wax sections were mounted on slides, which were dried for 30 min in an oven (60°C–70°C) and deparaffinized in xylene. The slides were then placed in changes of ethanol for 2 min each. Washing in buffer solution was performed between steps. The slides were then placed in 3% hydrogen peroxide for 15 min and subsequently incubated in avidin block for 15 min, biotin block for 15 min, primary antibody (Ang II-Antibody; Phoenix Pharmaceuticals Inc., Germany; 1 : 200) for 12 h at 4°C, and biotinylated secondary antibody for 1 h. The reagent incubation was performed with streptavidin peroxidase for 15 min. A 1-mi Mayer's hematoxylin counterstain was used. The slides were dehydrated, cleared with xylene, and mounted with permanent mounting medium. Finally, the pictures were analyzed by IPP 6.0 software.

### 2.8. Quantitative Real-Time PCR

The total mRNA of the renal cortical tissues of individual rats was extracted using TRIzol (Biotech, China). cDNA was synthesized from 2 *μ*g of total RNA using an RT kit (Roche) following the manufacturer's instructions. The podocyte-associated gene mRNA amount was assessed by quantitative real-time PCR (ABI Prism 7000) and normalized to the GAPDH. The sequences of the sense and antisense primers used for amplification are listed in Table 1.

### 2.9. Statistical Analysis

All data are presented as mean ± standard deviation (SD). Statistical analysis was carried out on three or more groups using one-way analysis of variance (ANOVA) and Dunnett's test. A value of *P* < 0.05 was considered statistically significant.

## 3. Results

### 3.1. Cardiac Function-Related Parameters

**Figure 1:**
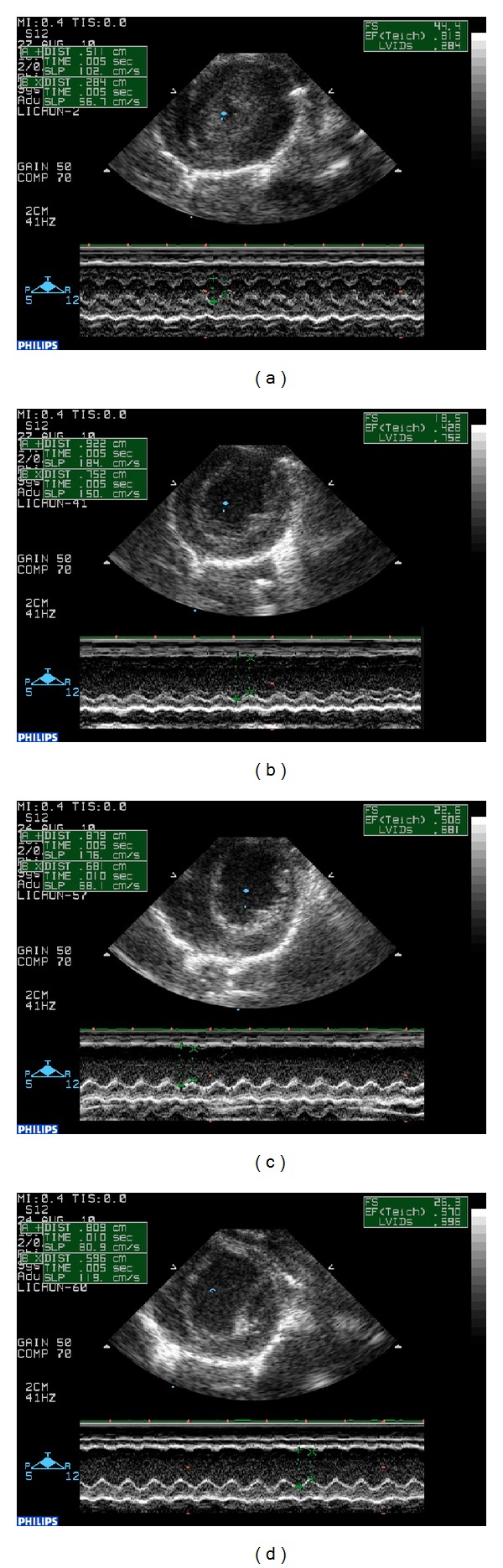
Cardiac function detected by echocardiography. (a) EF, FS, LVEDd, and LVEDs in sham-operated group. (b) Increased EF and FS and decreased LVEDd and LVEDs in sham-operated rats. (c) Changes in EF in fosinopril sodium group. (d) Improvements in EF and FS in QSYQ group.

At 28 days after surgery, echocardiography showed that EF and FS in the model group were significantly different (*P* < 0.05). The EF of the rats that underwent ligation in the model group decreased to 49.03% compared with that in the sham-operated group, and this decrease was accompanied by an increase in LVEDd and LVEDs, suggesting the development of cardiac hypertrophy in this stage. In the fosinopril sodium group, fosinopril sodium was shown to slightly improve the LVEDd and LVEDs, but no statistical significance was observed compared with the model group. Fosinopril sodium also upregulated the EF by 22.69%. After treatment with QSYQ for 28 days, the EF recovered by 37.62% compared with that in the model group. LVEDd and LVEDs were also lower than those of the model group (22.10% and 40.00%, resp.), but were still higher than those of the sham-operated group (Table 2, Figure 1).

### 3.2. Effects of QSYQ on Ang**  **II and Aldosterone

Plasma Ang II in the model group was higher than that in the sham-operated group (*P* < 0.05). After treatment with fosinopril sodium and QSYQ, Ang II decreased to 12.56% and 16.36%, respectively. The level of plasma aldosterone (Ald) in each group showed no significant difference (Table 3).

**Figure 2:**
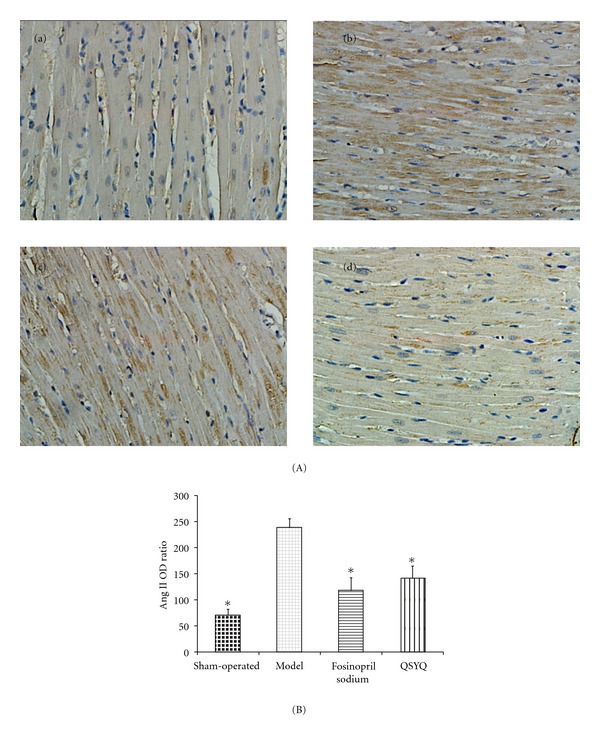
(A) Immunohistochemistry results in sham-operated, model, fosinopril sodium, and QSYQ groups (400x magnifications). (a) Cardiac Ang II expression in sham-operated group. (b) Upregulated cardiac Ang II expression in model group. (c) Fosinopril sodium reduced the level of Ang II. (d) Cardiac Ang II expression decreased in QSYQ group. (B) The OD ratio of cardiac Ang II expression. All values are means ± SD (*n* = 8). *P < 0.05 compared with model group.

Histological examination of the myocardial tissue via light microscopy showed that the levels of Ang II in the model group (238.45 ± 17.521) were upregulated compared with those in the sham-operated group (71.43 ± 10.439, *P* < 0.05). Fosinopril sodium (143.25 ± 23.977) downregulated the cardiac Ang II by its inhibitory effect on angiotensin-converting enzyme (ACE). In the QSYQ group, after treatment for 28 days, a reduction (122.33 ± 25.01) was detected compared with the model group (*P* < 0.05), which almost returned to the level of the sham-operated group but still showed a significant difference (*P* < 0.05) (Figure 2). This effect was similar to that observed in the fosinopril sodium group, suggesting almost equivalent efficacy on Ang II between the two groups.

### 3.3. Effects of QSYQ on ACE and ACE2

ACE and the more recently discovered ACE2 are important proteins involved in the RAAS pathway. The balance between ACE and ACE2 is important for the regulation of blood pressure and electrolyte homeostasis. The PCR results in our study showed that in rats with CHD, the ACE concentration (1.28 ± 0.045) was higher than that in the sham-operated group (0.93 ± 0.014, *P* < 0.05). As an ACE inhibitor (ACEI), fosinopril sodium (1.12 ± 0.126) can definitely decrease its level (1.28 ± 0.045, *P* < 0.05). QSYQ (1.23 ± 0.071) also can downregulate the ACE level to some extent, but showed no significance compared with the model group.

**Figure 3:**
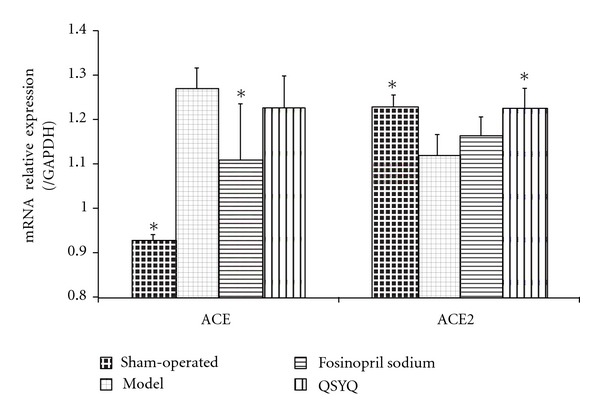
Cardiac ACE and ACE2 mRNA expression in rats. The relative levels of cardiac ACE and ACE2 mRNA were assessed by qPCR. Results were normalized to GAPDH. All values are means ± SD (*n* = 8). *P < 0.05 compared with model group.

ACE2 is a key negative regulator of RAAS, where it metabolizes Ang II into Ang 1–7, an endogenous antagonist of Ang II, producing a cardioprotective effect against CHD. In our study, ACE2 in the model group was downregulated (1.12 ± 0.048) compared with that in the sham-operated group (1.23 ± 0.027); after treatment with fosinopril sodium, it increased (1.17 ± 0.041), but no statistical difference was found compared with the model group. Interestingly, fosinopril sodium showed better efficacy in the QSYQ group (1.23 ± 0.045) in terms of upregulating ACE2 (Figure 3). 

### 3.4. Effects of QSYQ on Renin, AT1, and AT2

**Figure 4:**
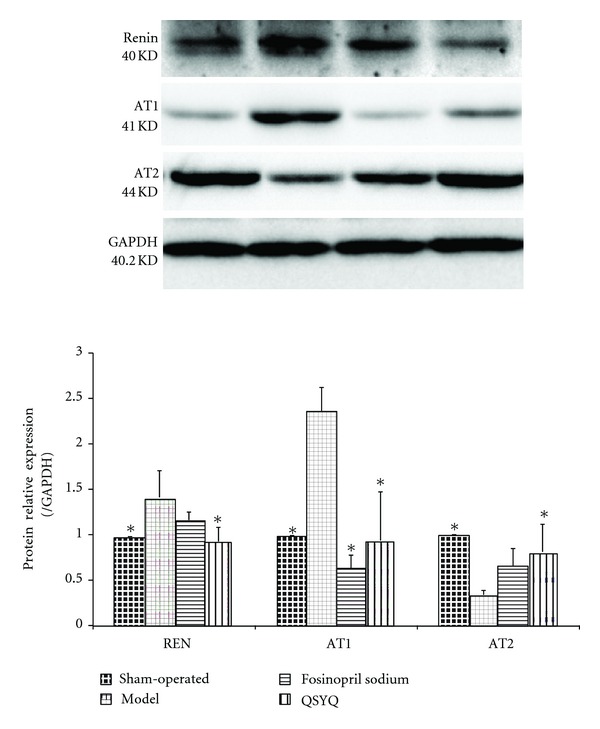
QSYQ significantly decreased cardiac renin and AT1 and increased AT2 in rats with CHD. Data were analyzed by one-way ANOVA, with *P* < 0.05 indicating statistical significance. *Differed significantly from the model group (*P* < 0.05).

 Western blot analysis of renin showed that at the end of the study, the cardiac renin in the model group (1.41 ± 0.292) increased compared with that in the sham-operated group (1.00 ± 0.000, *P* < 0.05). After treatment with fosinopril sodium (1.16 ± 0.087), the renin concentration was not significantly different between the groups; after treatment with QSYQ for 28 days, the level of renin (0.92 ± 0.154, *P* < 0.05) showed a 22.76% reduction compared with the model group, which had no statistical significance compared with the sham group (Figure 4). 

Two distinct subtypes of Ang II receptors mediate the predominant actions of RAAS [9]. AT1 is thought to be an ideal target for treatment of CHD [10]. In our study, AT1 in the model group was upregulated (2.38 ± 0.256) after surgery. In the fosinopril sodium group (0.65 ± 0.142), its level was suppressed. In the QSYQ group, the AT1 level (0.93 ± 0.535) decreased compared with that in the model group (*P* < 0.05), which had no significant difference compared with the sham-operated group (1.00 ± 0.000, *P* > 0.05) or fosinopril sodium group (Figure 4). 

AT2 has cardioprotective effects on attenuation of MI-induced impairments and caused a decrease in ventricular wall thinning [11]. In our study, AT2 in the model group was downregulated (0.33 ± 0.047) after surgery compared with that in the sham group (1.00 ± 0.000, *P* < 0.05). However, no difference was observed between the model and fosinopril sodium groups (0.65 ± 0.189, *P* > 0.05). In the QSYQ group, the AT2 level increased (0.81 ± 0.320) compared with that in the model group, but showed no significant difference compared with the sham-operated group (Figure 4).

### 3.5. Effects of QSYQ on MMP-9 and TGF-*β*

**Figure 5:**
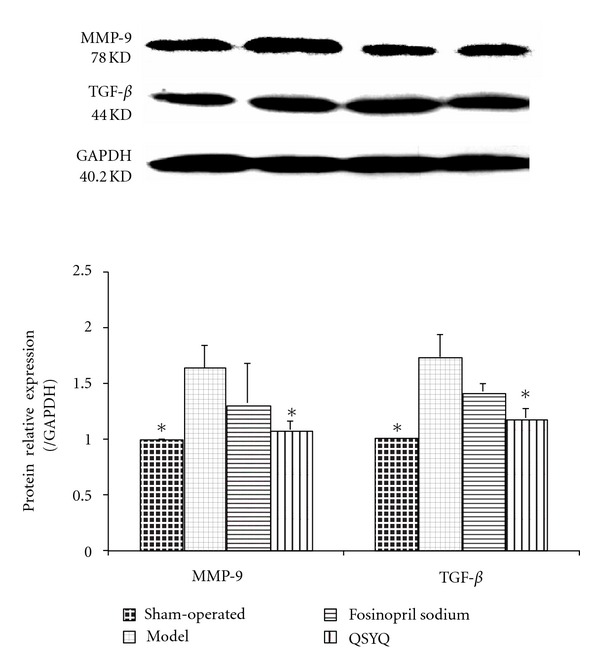
QSYQ significantly lowered cardiac MMP-9 and TGF-*β* in rats with CHD. Data were analyzed by one-way ANOVA, with *P* < 0.05 indicating statistical significance. *Differed significantly from the model group (*P* < 0.05).

The western blot of MMP-9 showed that at the end of the study, the cardiac MMP-9 in the model group increased (1.64 ± 0.266, *P* < 0.01) compared with that in the sham-operated group (1.00 ± 0.000). After treatment with fosinopril sodium (1.33 ± 0.354), the MMP-9 concentration was not significantly different from that in the model group, while after treatment with QSYQ, the level of MMP-9 was reduced compared with that in the model group (1.08 ± 0.077, *P* < 0.05) but showed almost no statistical significance when compared with the sham-operated group (Figure 5). 

Similar to MMP-9, in the model group, TGF-*β* was increased (1.74 ± 0.092, *P* < 0.01) compared with that in the sham-operated group (1.00 ± 0.000). After treatment with fosinopril sodium, the concentration (1.43 ± 0.069) showed no significant difference compared with that in the model group, while after treatment with QSYQ for 28 days, the level of TGF-*β* increased (1.19 ± 0.083) compared with that in the model group (*P* < 0.05). However, no statistical significance was observed when compared with the sham group (Figure 5).

## 4. Discussion

Chronic activation of RAAS is believed to contribute significantly to the deterioration of cardiovascular function. In this pathway, Ang II has a vast array of actions, including regulation of blood pressure, vasoconstriction, increasing aldosterone secretion, amplifying sympathetic activity, increasing sodium retention, and promoting cell growth and angiogenesis. It is considered to be a factor in virtually every form of CHD, and therapeutic strategies that block renin-angiotensin system activation using either ACEI or angiotensin receptor blockers favorably affect remodeling and reduce morbidity and mortality in post-MI and heart failure patients [12–14]. Among them, antagonists to AT1R and inhibitors of ACE have been routinely used to treat patients with CHD [6, 15]. Experimental studies have shown that ACEI, besides inhibiting the formation of Ang II, could have desirable effects by decreasing the breakdown of bradykinin [7]. Another important problem with long-term ACE inhibition is that after some time, plasma levels of Ang II return to pretreatment levels [16]. Because ACEI does not seem to offer complete protection against the detrimental effects of Ang II, AT1-receptor blockers may offer advantages relative to ACEI by effectively blocking the AT1-receptor, which mediates all known harmful effects of Ang II [17]. Recently, the subtypes of both ACE and AT1 were identified: ACE2 and AT2. They have distinct roles with ACE and AT1. ACE2 is thought to be a key negative regulator of the renin-angiotensin system, where it metabolizes Ang II into Ang1–7, an endogenous antagonist of Ang II. Both the prolonged activation of RAAS and the loss of ACE2 can be detrimental because they lead to functional deterioration of the heart and progression of cardiac, renal, and vascular diseases [11]. Loss of ACE2 in post-MI mice is associated with increased Ang II levels and ROS production. This is followed by increased MMP activation and activation of TGF-*β* in ACE2-deficient hearts [18], thus causing clinical cardiac hypertrophy. Moreover, AT2 is highly expressed in the developing fetus, but its expression in the cardiovascular system is low and declines after birth. However, the expression of AT2 appears to be modulated by pathological states such as hypertension, MI, or any pathology associated with tissue remodeling [19]. In the posttreatment study, the overexpression of AT2R partly reversed the MI-induced cardiac dysfunction. MI also induced the upregulation of Ang II type 1 receptor, ACE, and collagen I mRNA expression, all of which are attenuated by the overexpression of AT2R [20]. In the failing heart, AT2R is reexpressed in cells proliferating in interstitial regions or neointima and exerts an inhibitory effect on Ang II-induced extracellular matrix proteins, resulting in attenuation of tissue remodeling. AT2R also activates the nitric oxide/cGMP system in cardiovascular tissue, resulting in AT2R-mediated cardioprotection and vasodilation [21].

In our research, QSYQ significantly downregulated the level both Ang II and AT1R, indicating efficacy similar to that of AT1 agonists. In addition, it upregulated the AT2 level to inhibit MMP-9 and TGF-*β*, thus providing a cardioprotective effect, while AT1 does not have similar efficacy. Although QSYQ has no effect on ACE, unlike fosinopril sodium, it has an effect on another subtype (ACE2) and likely reduces the MMP-9 level by the TGF-*β*-NF-*κ*B pathway [22]. This suggests safe and complementary application to clinical CHD therapy.

 Interestingly, QSYQ can lower the RAAS activation from the beginning via renin. Renin is an aspartyl-protease enzyme produced and activated within the juxtaglomerular cells of the afferent arterioles in the kidney. Through Ang I, it can activate Ang II, which is the primary biologically active hormone of the RAAS. Renin secretion is the critical rate-limiting step in the entire system [19]. Therefore, the regulation of renin secretion by QSYQ is of particular interest and importance in understanding its collaborative effect with Ang II as well as understanding the therapeutic targets for CHD. Ald seems to remain unchanged, which is consistent with the published papers [23]. “Ald breakthrough” is thought to be an important mechanism.

In summary, this paper presents a study of multitargets for a Chinese herbal formula. The results are consistent with a report showing that known human drug targets tend to maintain a balance between injury and protective aspects [24]. TCM with multiple chemical components targets multiple proteins, which may produce greater synergetic efficacy and fewer side effects. The present results also show that QSYQ can act on CHD by different targets of RAAS, especially renin and Ang II, ACE/ACE2, and AT1/AT2, eventually decreasing the levels of the MMP-9 and TGF-*β*, which can treat CHD efficiently and safely. 

## Figures and Tables

**Table 1:** Nucleotide sequences of primers used in real-time PCR.

Gene (accession no.)	Primers	Nucleotide sequence 5′-3′	Size (bp)	Temp. (°C)
ACE	Forward	GTCCTATTCCCGCTCATCT	128	53.1
	Reverse	CCAGCCCTTCTGTACCATT		

ACE2	Forward	AGAATGCGACCATCAAGCG	230	52.6
	Reverse	AAGCCCAGAGCCTACGATT		

GAPDH	Forward	CACTGCCACTCAGAAGACT	177	53.5
	Reverse	ACGTTGGGGGTAGGAACAC		

**Table 2:** Echocardiography results of rats in each group.

Group	*N *	LVEDd/cm	LVEDs/cm	FS/%	EF/%
Sham-operated	8	0.65 ± 0.104*	0.37 ± 0.128*	43.92 ± 9.048*	81.52 ± 5.968*
Model	8	0.95 ± 0.104^▲^	0.77 ± 0.134^▲^	25.33 ± 11.176^▲^	41.55 ± 14.371^▲^
Fosinopril sodium	8	0.89 ± 0.140^▲^	0.70 ± 0.133^▲^	24.20 ± 9.285	50.98 ± 9.094^▲∗^
QSYQ	8	0.74 ± 0.130^▲∗^	0.55 ± 0.141^▲∗^	29.71 ± 9.993^▲^	57.18 ± 11.678^▲∗^

^▲^ *P* < 0. 05, versus sham-operated group; *P < 0.05, versus model group.

**Table 3:** Concentration of Ald and Ang II in plasma.

Group	*N *	Ang II (×10^−6^ *μ*g/mL)	ALD (×10^−3^ *μ*g/mL)
Sham-operated	8	165.59 ± 21.352*	208.85 ± 47.953
Model	8	211.28 ± 19.853^▲^	220.32 ± 20.608
Fosinopril sodium	8	184.75 ± 29.096*	228.72 ± 17.603
QSYQ	8	176.71 ± 27.661*	236.49 ± 32.965

^▲^ *P* < 0.05 versus sham-operated group; *P < 0.05 versus model group.
