# Corrigendum to “Cardioprotective Effects of Qishenyiqi Mediated by Angiotensin II Type 1 Receptor Blockade and Enhancing Angiotensin-Converting Enzyme 2”

**DOI:** 10.1155/2019/9791436

**Published:** 2019-01-10

**Authors:** Yong Wang, Chun Li, Yulin Ouyang, Junda Yu, Shuzhen Guo, Zhongyang Liu, Dong Li, Jing Han, Wei Wang

**Affiliations:** ^1^Beijing University of Chinese Medicine, Bei San Huan Dong Lu 11, Chao Yang District, Beijing 100029, China; ^2^State Key Laboratory of Proteomics, Beijing Proteome Research Center, Institute of Radiation Medicine, Beijing 100850, China

In the article titled “Cardioprotective Effects of Qishenyiqi Mediated by Angiotensin II Type 1 Receptor Blockade and Enhancing Angiotensin-Converting Enzyme 2” [1], the wrong TCM remedy was cited in the Introduction. The article focuses on the pharmacological effect of Qishenyiqi (QSYQ) on coronary heart disease, but two articles about Qishenyiqi drop pill, which has a similar name and the same abbreviation, QSYQ, were inappropriately discussed as references 2 and 3. Both remedies include Radix Astragali mongolici (‘huang-qi' in Chinese) and Salvia miltiorrhiza bunge (‘dan-shen' in Chinese).

Therefore, references 2 and 3 and the following wording should be removed from the introduction: “is widely produced in China in accordance with the China pharmacopoeia standard of quality control [2]. It is commonly used in routine treatment of CHD in clinical practice in China. It contains large-scale epidemiological survey in the randomized, controlled clinical trials proved that it has a definite effect on improving heart function [3].” Accordingly, the first sentence in the first paragraph of the Introduction should read as follows:

“The ancient TCM Qishenyiqi (QSYQ) was prepared from a basic formula of six Chinese herbs (Radix Astragali Mongolici, salvia miltiorrhiza bunge, Flos Lonicerae, scrophularia, Radix Aconiti Lateralis Preparata, and Radix Glycyrrhizae).”

## References

[1] Wang Y., Li C., Ouyang Y. (2012). Cardioprotective effects of qishenyiqi mediated by angiotensin II type 1 receptor blockade and enhancing angiotensin-converting enzyme 2. *Evidence-Based Complementary and Alternative Medicine*.

